# Microbiome–Metabolomics Analysis Insight into the Effects of Starvation and Refeeding on Intestinal Integrity in the Juvenile Largemouth Bass (*Micropterus salmoides*)

**DOI:** 10.3390/ijms252312500

**Published:** 2024-11-21

**Authors:** Zhenxin Zhao, Xianbo Zhang, Fei Zhao, Tianxun Luo

**Affiliations:** 1 Institute of Fisheries, Guizhou Academy of Agricultural Sciences, Guiyang 550025, China; xianbozhang1991@126.com (X.Z.); zhaofei8@163.com (F.Z.); lutaxo@gmail.com (T.L.); 2 Guizhou Special Aquatic Products Engineering Technology Center, Guiyang 550025, China

**Keywords:** aquaculture, feeding, gene expression, intestinal microbiota, oxidative stress, antioxidant defenses

## Abstract

The effects of starvation and refeeding on the gut condition of juvenile largemouth bass (*Micropterus salmoides*) remain unclear. Therefore, our research aimed to explore these effects. Amylase and lipase activities were remarkably decreased in the starvation (ST) group, yet prominently increased in the refeeding (RE) group (*p* < 0.05). In addition to the malondialdehyde (MDA) level, catalase (CAT) and superoxide dismutase (SOD) activities were significantly upregulated in the ST group (*p* < 0.05) in marked contrast to those in the controls; however, the RE group showed no substantial variations in CAT and SOD activities or the MDA level (*p* > 0.05). During starvation, the expression of Nrf2-Keap1 pathway-associated genes was significantly upregulated (*p* < 0.05). The comparative levels of TNF-α, IL-1β, and IL-15 were highly increased, with the levels of TGF-β1 and IL-10 apparently downregulated in the ST group; in contrast, these levels were restored to their original values in the RE group (*p* < 0.05). In contrast to the controls, the ST group showed significantly lower height and width of the villi, muscle thickness, and crypt depth and a higher goblet cell number; however, these values were recovered to some extent in the RE group (*p* < 0.05). The dominant bacterial phyla in the intestines of both groups were Proteobacteria, Firmicutes, Bacteroidetes, Acidobacteria, and Actinobacteria, with marked inter-group differences in the genera *Serratia* and *Lactobacillus*. Metabolomics analysis showed that amino acid metabolism is disrupted during starvation and is restored after refeeding. In summary, this study expands our comprehension of the interaction between oxidative stress and antioxidant defenses among juvenile largemouth bass subjected to starvation and refeeding.

## 1. Introduction

Aquatic animals often encounter various stresses while living in their habitats. One of the frequent and severe adversities is intermittent food restriction (FR) in the farm or habitat due to migration, reproduction, feed shortage, and variability in rhythmicity and dietary habits, particularly in winter [1] (Morshedia et al., 2017). The prolonged duration of starvation or FR can invariably trigger alterations in biochemical and physiological responses in aquatic animals, potentially leading to weight loss, depleted energy reserves, changes in metabolic function, reduced swimming skills, and even death [2] (MacDonald et al., 2018). Fish species are considered to be particularly vulnerable to starvation, indicating that their low resistance to food deprivation is a more significant issue than that of other mammals. Hence, it is beneficial to assess biochemical and physiological modifications in aquatic animals under food scarcity conditions, which could enable the development of optimal feeding strategies.

The intestinal tract is considered an essential physiological and immune system component. It constitutes 70% of the immune system’s barrier function between the body and the external environment and nutrient absorption [3,4] (Chen et al., 2020; Winer et al., 2016). Thus, intestinal dysfunction may impair antioxidant function and systemic immunity, thereby decreasing growth performance and nutrient absorption [5,6,7] (Cheng et al., 2019; Citi et al., 2018; Ulluwishewa et al., 2011). Intestines are sensitive to food restriction, and the gut health status rapidly changes following starvation. A potential impact of nutritional stress is that it interferes with nutrient absorption and digestion and disrupts intestinal processes vital for energy provision and the maintenance of basic survival functions [8] (Caruso et al., 2014). Prior research has illustrated that fish are capable of altering their intestinal immune and antioxidant functions to address starvation [9] (Zhao et al., 2022) and modulating the activities of enzymes associated with digestion [10] (Mogensen et al., 2012). For instance, in *Trachinotus mookalee*, the activities of amylase, chymotrypsin, trypsin, and pepsin were reduced when food was restricted but returned to normal levels upon refeeding [11] (Xavier et al., 2023). Conversely, the gut activities of catalase (CAT), superoxide dismutase (SOD), glutathione peroxidase (GPx), and glutathione S-transferase appeared to gradually increase under food deprivation but apparently decreased after the restoration of food supply in *Catla catla* (Pal et al., 2018) [12]. Similarly, starvation markedly induces oxidative stress (OS), leading to a remarkable elevation in the expression levels of SOD and CAT in *Litopenaeus vannamei* (Lin et al., 2012) [13], *Labeo rohita* (Dar et al., 2019) [14], and *Amphiprion melanopus* (Choi et al., 2012) [15]. Conversely, among fish with an increase in starvation stress, the functional downregulation of the intestine is accompanied by substantial disruption of microbiota composition. The intestinal flora regulates metabolic processes and immune responses to maintain energy homeostasis [16] (Johnson et al., 2018). Variations in the intestinal microbial community of fish affect their immunoregulation and nutrient uptake [17,18] (Zhao et al., 2023; Mekuchi et al., 2018). According to previous studies on *Gadus morhua* L. (Dhanasiri et al., 2011) [19], *Lates calcarifer* (Xia et al., 2014) [20], and *Ctenopharyngodon idellus* (Tran et al., 2018) [21], food restriction influences not only immune activity but also the community structures of the gut microbiota. Therefore, it is essential to investigate how fish species differ in their intestinal digestive functions, immune characteristics, and intestinal microbiota in response to nutritional stress.

The largemouth bass *Micropterus salmoides* is famous for its swift development, delectable flavor, robust disease resistance, and significant economic and nutritional value. It is widely cultivated and has emerged as a crucial aquatic food source in China [22] (Cao et al., 2024). China produced a total of 802,486 tons of cultivated products in 2022, 702,093 tons in 2020, and 619,519 tons in both 2021 and 2022 [23] (Yearbook, 2021–2023). Food restrictions, which can be caused by water temperature, oxygen levels, food fluctuations, and improper feeding methods, are among the most significant hazards to aquaculture. Nevertheless, the influence of hunger stress on the intestinal health of largemouth bass remains unknown. Hence, our research analyzed the effect of food deprivation and refeeding on intestinal morphology, digestion, immunity, and microbial community structure of largemouth bass. This study’s findings can enhance our understanding of feeding methods and thereby improve the management of largemouth bass in the domain of aquaculture.

## 2. Results

### 2.1. Comparison of Digestive/Antioxidant Enzyme Activities

**Figure 1:**
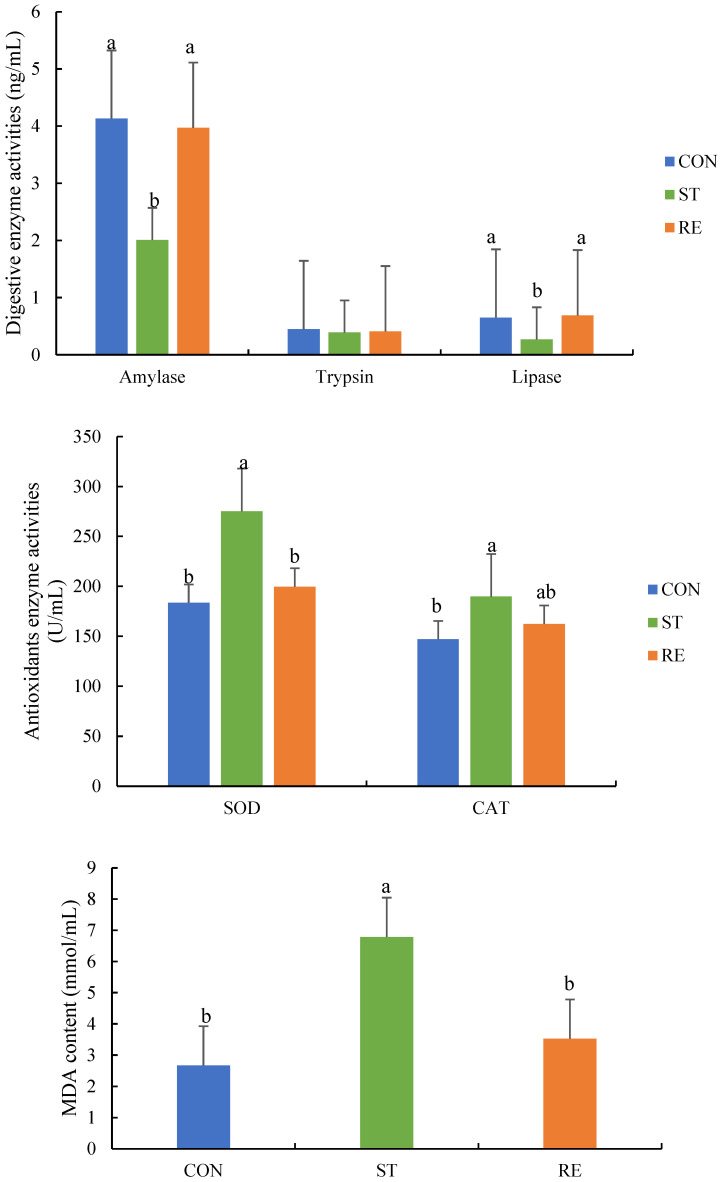
Digestive and antioxidant enzyme activities of juvenile largemouth bass subjected to starvation and refeeding at the end of the experiment. Note: Values are means ± SEM. Different letters denote significant differences (*p* < 0.05). SOD: superoxide dismutase, CAT: catalase, MDA: malondialdehyde.

As shown in Figure 1, lipase and amylase activities were markedly elevated in the ST group compared with those in the CON and RE groups (*p* < 0.05). Nonetheless, these two groups exhibited no discrepancies in lipase and amylase activities (*p* > 0.05). Moreover, the trypsin levels did not differ remarkably among all groups (*p* > 0.05). In addition to the MDA level, CAT and SOD activities were prominently upregulated by more than 1.3-fold in the ST group compared to those in the CON group (*p* < 0.05). After refeeding, SOD and CAT activities and the MDA level of the RE group were comparable to those in the CON group (*p* > 0.05).

### 2.2. Expression Levels of Antioxidant Genes

**Figure 2:**
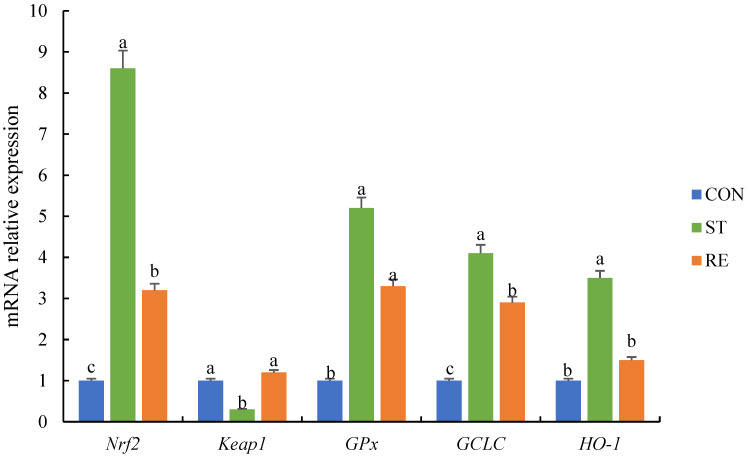
qRT-PCR analysis of the expression of Nrf2-Keap1 pathway-related genes in the anterior intestine of juvenile largemouth bass after the onset of starvation and refeeding. Note: Values are means ± SEM. Different letters denote significant differences (*p* < 0.05). *Nrf2*: nuclear factor erythroid 2-related factor 2, *Keap1*: Kelch-like ECH-associated protein 1, *GPx*: glutathione peroxidase, *GCLC*: glutamate-cysteine ligase catalytic subunit, *HO-1*: heme oxygenase-1.

Figure 2 indicates that starvation stress markedly upregulated the expression levels of genes regarding the Nrf2-Keap1 axis, including *HO-1*, *GCLC*, *GPx*, and *Nrf2*, while reducing the expression of *Keap1* (*p* < 0.05). The expression levels of *HO-1* and *Keap1* returned to normal values following refeeding (*p* < 0.05).

### 2.3. Expression Levels of Inflammation-Associated Factors

**Figure 3:**
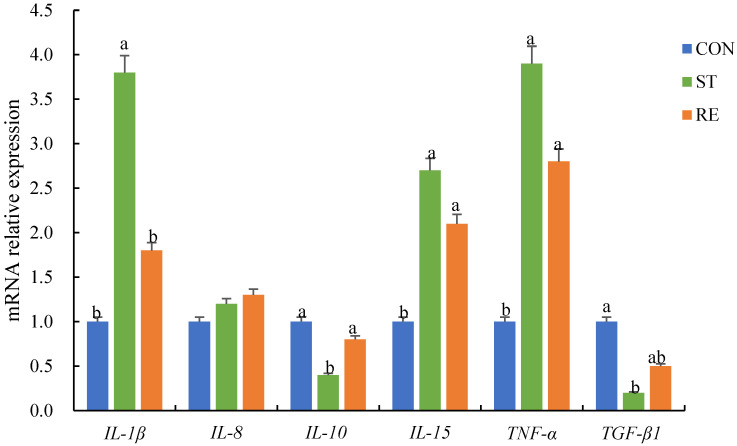
qRT-PCR analysis of the expression of inflammation-related genes in the anterior intestine of juvenile largemouth bass after the onset of starvation and refeeding. Note: Values are means ± SEM. Different letters denote significant differences (*p* < 0.05). *IL-1β*: interleukin-1β, *IL-8*: interleukin-8, *IL-10*: interleukin-10, *IL-15*: interleukin-15, *TNF-α*: tumor necrosis factor, *TGF-β1*: transforming growth factor-β1.

The expression levels of inflammation-related factors in different groups were measured (Figure 3). During the starvation process, the yield of *TNF-α*, *IL-1β*, and *IL-15* was elevated, whereas those of *TGF-β1* and *IL-10* was markedly downregulated compared to those in the CON group (*p* < 0.05). After refeeding, the *IL-1β* level decreased relative to that in the ST group (*p* < 0.05), while the levels of *TGF-β1*, *IL-10*, and *IL-1β* equated to those of the CON group (*p* < 0.05). The *IL-8* level was comparable among the CON, ST, and RE groups (*p* > 0.05).

### 2.4. Histological Structure of the Intestine

**Figure 4:**
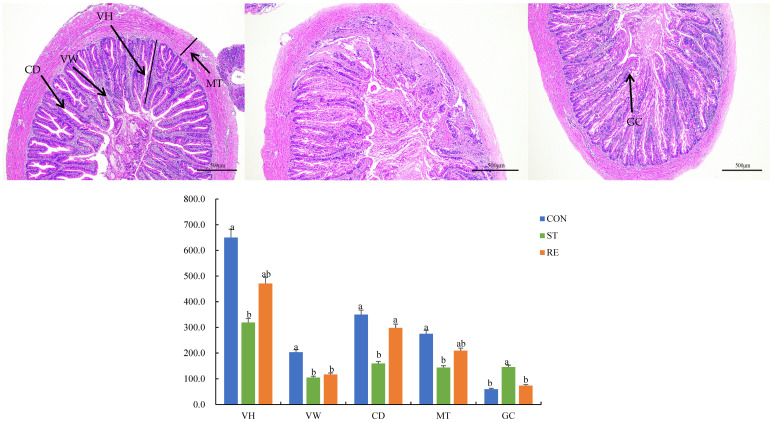
Histological examination the mid-gut morphology after the onset of starvation and refeeding using H&E and AB-PAS staining in juvenile largemouth bass. VH: villus height (µm), VW: villus width (µm), MT: muscle thickness (µm), CD: crypt depth (µm), GC: goblet cells. Note: Values are means ± SEM. Different letters denote significant differences (*p* < 0.05).

Figure 4 shows intestinal morphology after starvation and refeeding. The villi height (VH), villi width (VW), muscle thickness (MT), and crypt depth values were markedly decreased in the ST group, in stark contrast with those in the CON group. Moreover, there was a tendency for a progressive quantity of goblet cells (GCs) in the ST group (*p* < 0.05). On the contrary, no remarkable differences in the VH, VW, and MT values were particularly noteworthy between the ST and RE groups (*p* > 0.05).

### 2.5. Intestinal Microbiota Analysis

**Figure 5:**
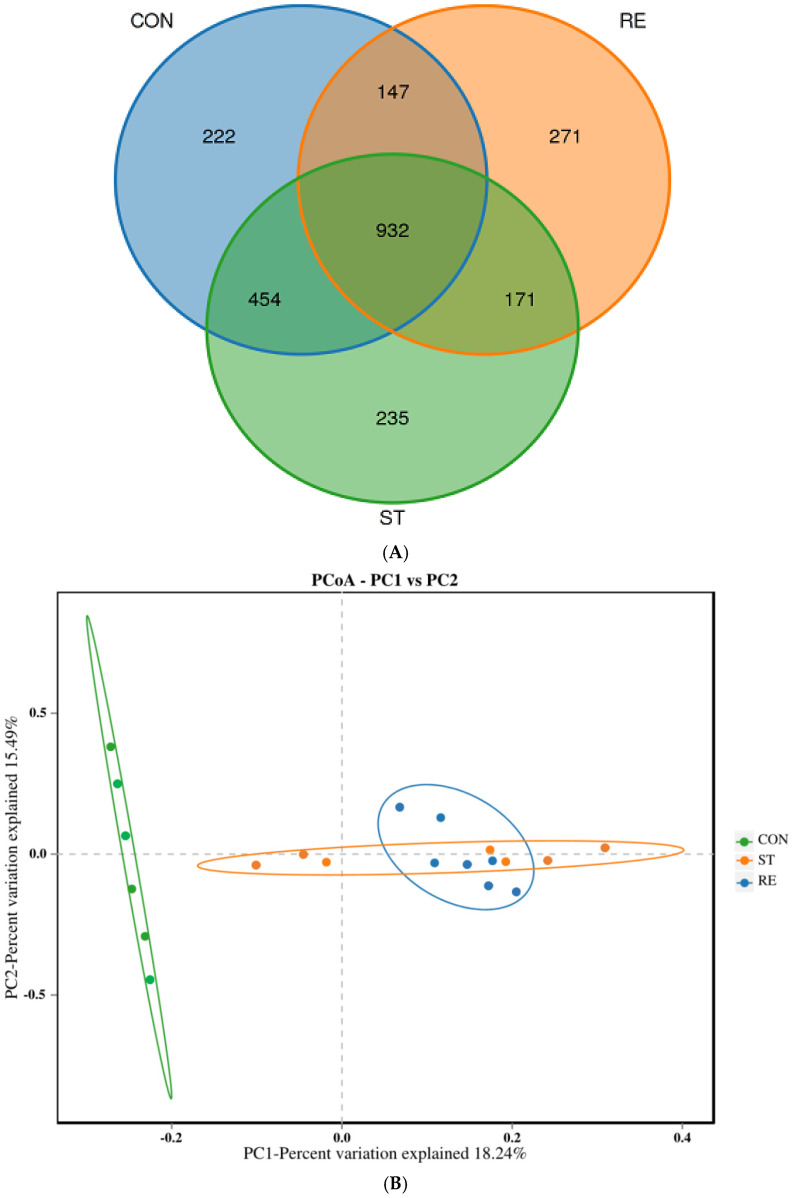

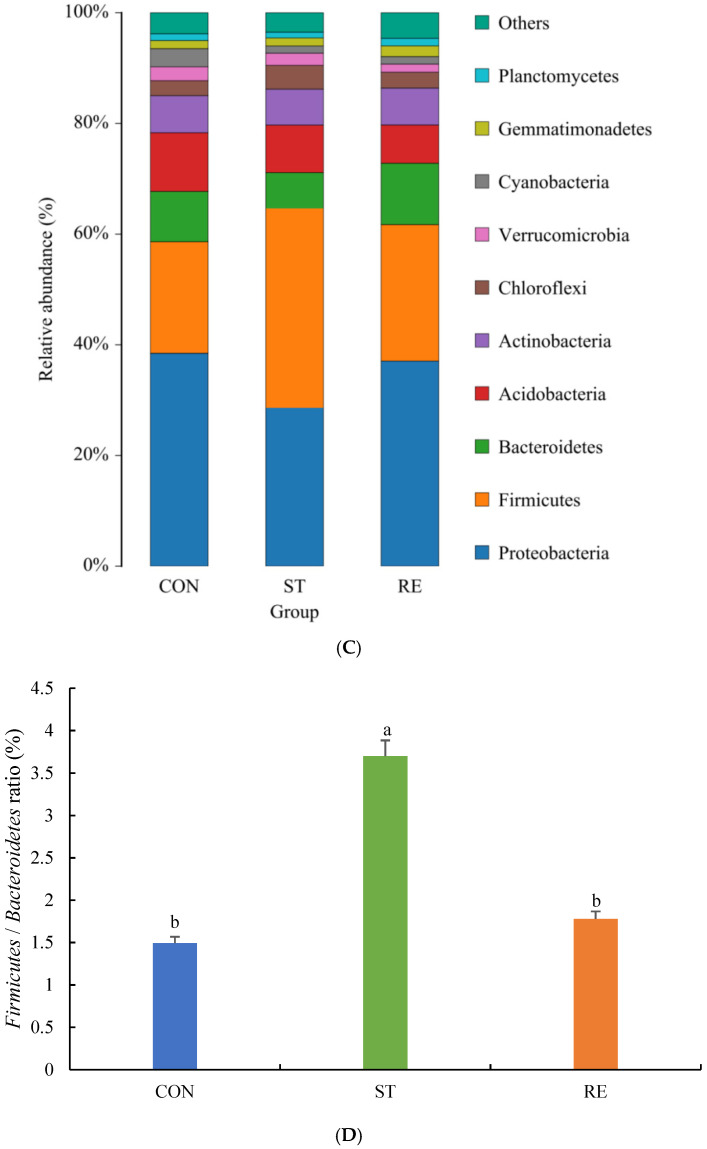

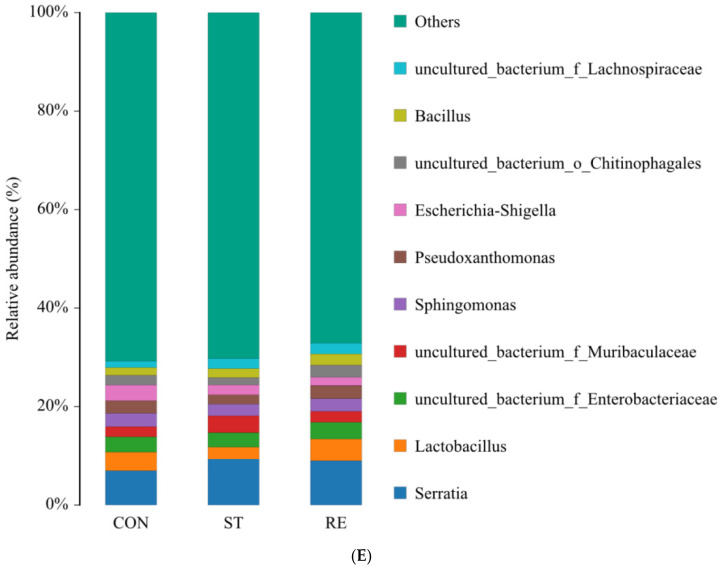
The effects of starvation and refeeding on 16S rRNA gene sequencing of the intestinal microbiota in juvenile largemouth bass (*n* = 6 per group). (**A**) Venn diagram; (**B**) PCA diversity; (**C**) Alterations in microbiota at the phylum level; (**D**) The abundance of the Firmicutes/Bacteroidetes (F/B) ratio in different groups, diverse lowercase letters above the bars show significant differences (*p* < 0.05) in different groups; (**E**) Alterations in microbiota at the genus level.

With respect to juvenile largemouth bass in disparate groups, the features of the gut microbiota were recognized through 16S rRNA sequencing. In total, 226,026 effective readings were acquired, representing samples showing efficient rates and Q30 values exceeding 93.61% and 93.88%, respectively (Table 1). Starvation stress and refeeding had significant effects on the diversity (Shannon index) and richness (Chao1 index), respectively (*p* < 0.05; Table 2). Similarities and overlaps between the operational taxonomic units (OTUs) of the different groups are shown in the Venn diagram (Figure 5A). Overall, 932 common OTUs were found among the three groups. The CON, ST, and RE groups had 222, 235, and 271 unique OTUs, respectively. Regarding β-diversity, principal coordinate analysis (PCoA) revealed that the clustering of the microbiota in the ST and RE groups differed from that in the CON group (Figure 5B). Figure 5C displays the microbial abundance and community composition at the phylum level. Acidobacteria, Actinobacteria, Bacteroidetes, Firmicutes, and Proteobacteria were the predominant phyla among all three groups. Proteobacteria and Bacteroidetes showed the lowest abundance, and Firmicutes exhibited the maximal abundance in the ST group compared to those in the remaining groups; the proportion of Firmicutes to Bacteroidetes (F/B) was markedly higher in the ST group (Figure 5D). Furthermore, as shown in Figure 5E, the dominant bacterial genera were *Serratia*, *Lactobacillus*, *uncultured_bacterium_f_Enterobacteriaceae*, *Pseudoxanthomonas*, *Sphingomonas*, and *uncultured_bacterium_f_Muribaculaceae*. *Lactobacillus* showed a lower proportion in the ST group compared to the other groups.

**Figure 6:**
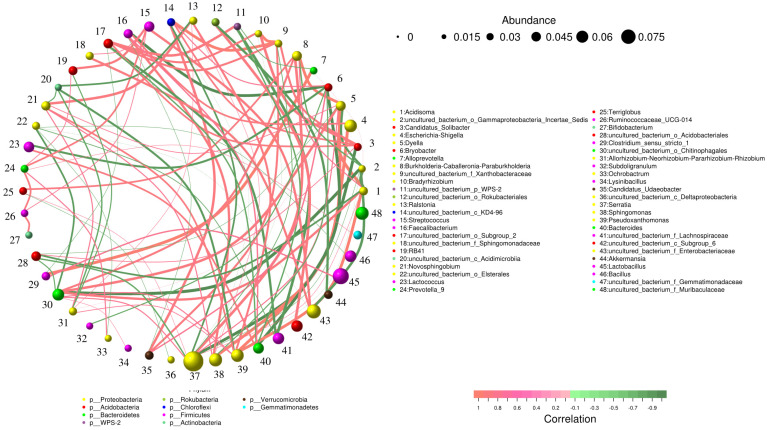
Spearman’s correlation-based network analysis of bacterial communities for interactions among genera. Nodes (colored dots) represent genera, whose size is proportional to their relative abundance. A line between two nodes indicates a significant positive (orange, Spearman’s correlation, *rs* > 0.1 and *p* < 0.05) or negative (green, Spearman’s correlation, *rs* > −0.1 and *p* < 0.05) correlation, with its thickness reflecting the relative strength.

In order to deeply explore the underlying relationships between “collaborative” or “competing” microbiota within the rhizosphere, correlation coefficients were estimated using Spearman’s rank method between the most prevalent species (Figure 6). The results showed correlations among the 48 most abundant genera, and the most closely associated genera were *Alloprevotella* and *Clostridium*_sensu_stricto_1.

**Figure 7:**
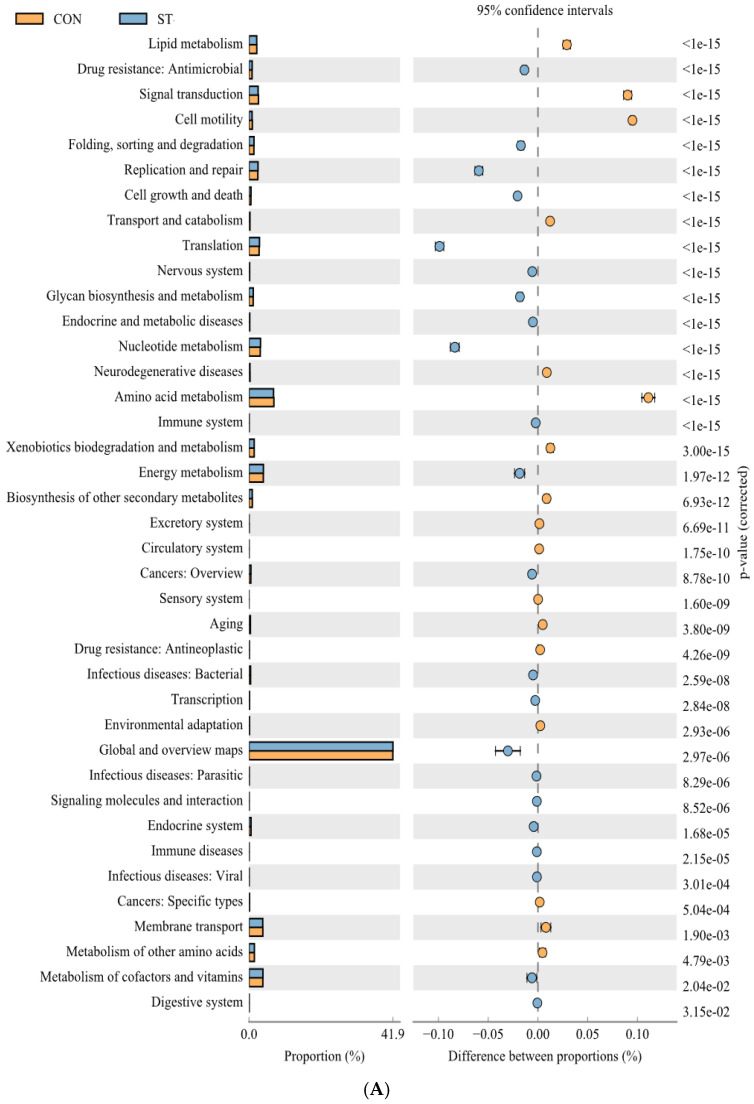

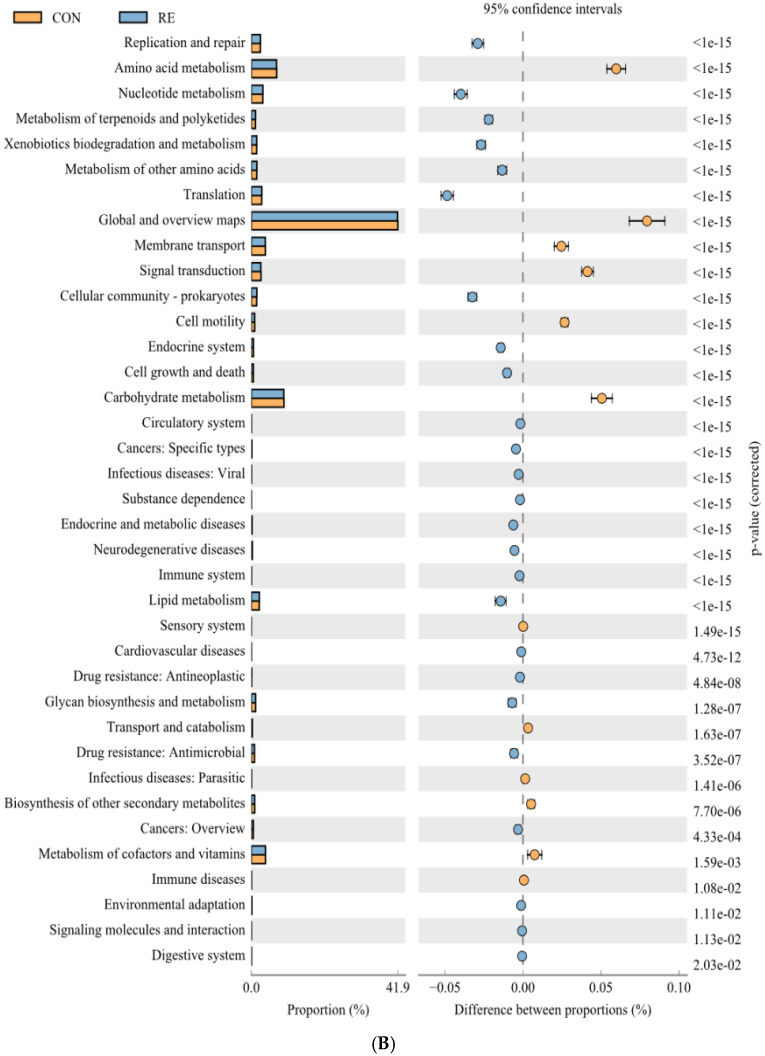
The functional pathways showing the highest differences between the (**A**) CON and ST, and (**B**) CON and RE using PICRUSt2 analysis, generated by the STAMP program. Bars on the left indicate the percentage of each category at different levels. Only categories with Bonferroni-corrected *p*-values exhibiting statistical differences at *p* < 0.05 are shown.

As shown by PICRUSt2 (Figure 7A,B), the predicted pathways differed significantly between the ST and RE groups relative to those in the CON group. The identified pathways were predominantly associated with processes such as translation, energy, and metabolism of lipids, nucleotides, carbohydrates, cofactors, and vitamins.

### 2.6. Intestinal Metabolomics Analysis

**Figure 8:**
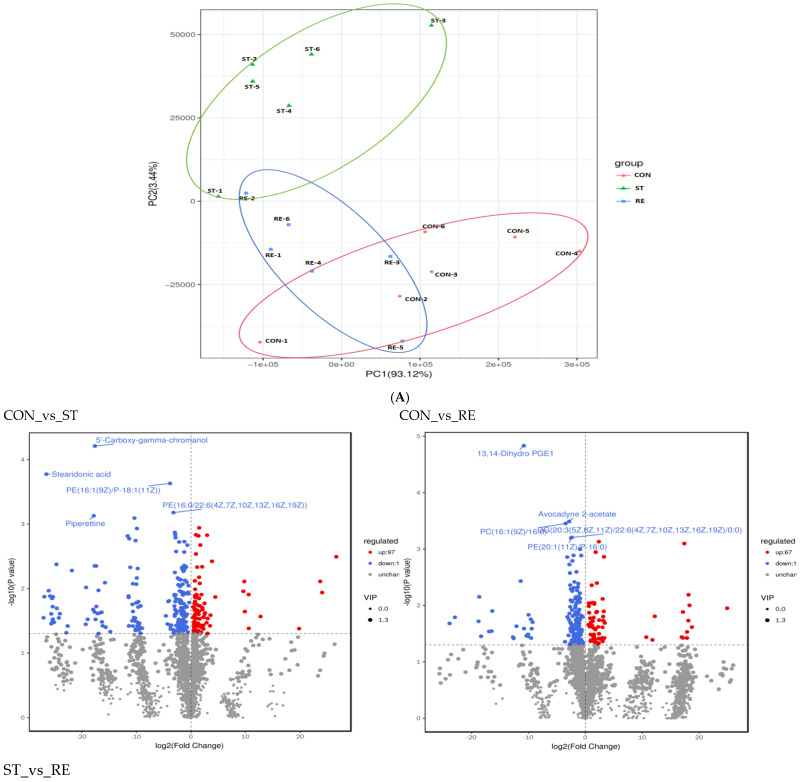

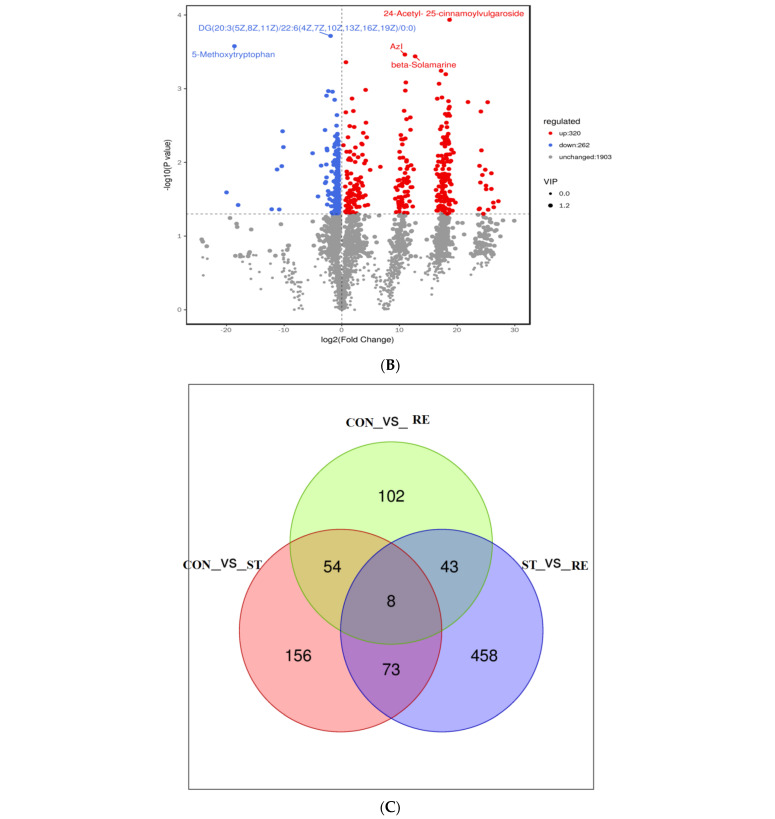
The effects of starvation and refeeding on the intestinal metabolic profile in juvenile largemouth bass (*n* = 6 per group). (**A**) PCA scores; (**B**) Volcano plot; (**C**) Venn analysis.

To determine metabolic changes induced by food restriction and refeeding in juvenile largemouth bass, we analyzed the metabolic profile of intestinal contents in the CON, ST, and RE groups. Consequently, PCA showed a distinct separation between the samples of the CON and RE groups compared to the ST group (Figure 8A). In order to recognize the differentially accumulated metabolites (DAMs), the contributions of candidate markers between two groups (Figure 8B)are shown through a volcano plot. There were 291, 207, and 582 DAMs from the “CON_vs_ST”, “CON_vs_RE” and “ST_vs_RE” comparisons, respectively. The biomarkers 5′-carboxy-gamma-chromanol, stearidonic acid, PE(16:1(9Z)/P-18:1(11Z)), PE(16:0/22:6(4Z,7Z,10Z,13Z,16Z,19Z)), and piperettine were chosen for the CON_vs_ST groups. The biomarkers 13,14-dihydro PGE1, avocadyne 2-acetate, PC(16:1(9Z)/16:0), DG(20:3(5Z,8Z,11Z)/22:6(4Z,7Z,10Z,13Z,16Z,19Z)/0:0), and PE(20:1(11Z)/P-16:0) were chosen for the CON_vs_RE groups. The biomarkers 24-acetyl-25-cinnamoylvulgaroside, DG(20:3(5Z,8Z,11Z)/22:6(4Z,7Z,10Z,13Z,16Z,19Z)/0:0), AzI, 5-methoxytryptophan, and beta-solamarine were chosen for the ST_vs_RE groups. Furthermore, for all 894 DAMs identified in the Venn diagram, 156, 102, and 458 DAMs were analyzed only in the CON_vs_ST, CON_vs_RE, and ST_vs_RE groups, respectively. Eight DAMs were simultaneously detected across the three groups (Figure 8C).

**Figure 9:**
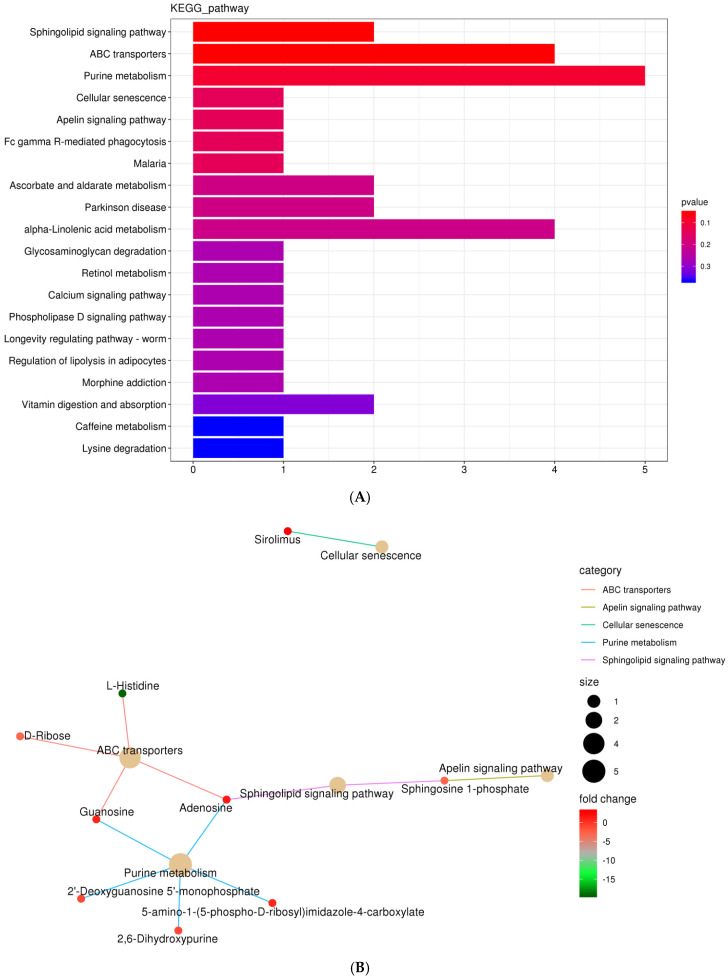
The KEGG functional annotation of significantly differential metabolites between the groups CON and ST. (**A**) Classification diagram. The horizontal axis is the number of different metabolites annotated to the pathway, and the vertical axis is the pathway name; (**B**) Network diagram. Note: The light-yellow nodes in the figure are the pathways, and the small nodes connected to each are the specific metabolites annotated to that pathway. The depth of the color indicates the difference multiple based on the log2 value.

Twenty metabolic biomarkers were distinctly different between the CON and ST groups, with the majority of the differentially enriched metabolites involved in purine metabolism, ABC transporters, and alpha-linolenic acid metabolism (Figure 9A). Among these pathways, the purine metabolism possessed the most annotated metabolites, such as adenosine, 2′-deoxyguanosine-5′-monophosphate, guanosine, and 5-amino-1-(5-phospho-D-ribosyl)imidazole-4-carboxylate (Figure 9B).

## 3. Discussion

The activity of digestive enzymes becomes a credible measure of nutrient status and digestive level in animals [24] (Bolasina et al., 2007). Research has discovered that food restriction, as an external stress factor, affects the activity of digestive enzymes (lipase, pepsin, amylase, etc.) to mobilize the energy reserves from the intestine, and these activities reach basal levels by refeeding in a few fish species [11] (Xavier et al., 2023). Former research has proven that the digestive enzyme functions of Atlantic cod [25] (Belanger et al., 2002), Atlantic salmon [26] (Krogdahl et al., 2005), and Rohu (Yengkokpam et al., 2013) [27] decreased rapidly during starvation but increased considerably after refeeding. This research found that lipase and amylase activities were markedly reduced with starvation and recovery compared to that of controls following refeeding. These findings corresponded to those of Abolfathi et al. (2012) [28], who discovered a marked decrease in the amylase activity of juvenile roaches under starvation stress followed by refeeding. This indicates that during refeeding, the recovery of digestive enzyme activities is prioritized (Xavier et al., 2023) [11]. Conversely, in-depth research is indispensable for expounding the mechanisms through which starvation and refeeding influence the OS and antioxidant responses of juvenile largemouth bass.

The intestinal antioxidant status is important for sustaining the integrity of the gut structure, which influences nutrient uptake and works as a necessity for piscine development (Zhao et al., 2014) [29]. However, as a stressor, starvation might produce oxidative damage, leading to a reduction in antioxidant content and the generation of oxygen free radicals, leading to intestinal protein peroxidation and lipid oxidation injury (Morales et al., 2004; Jiang et al., 2016) [30,31]. Noticeably, the intestines of an aquatic animal possess a physiological system to alleviate OS; this system comprises nonenzymatic antioxidants and enzymatic ones (e.g., CAT and SOD) (Biller et al., 2018; Wang et al., 2020) [32,33]. Our research unveiled that the activities of CAT and SOD were obviously increased during starvation. However, their activity was reduced after 7 days of refeeding. Furné et al. (2009) [34] also found increased SOD and CAT activities with food restriction and a decline in these enzyme activities after refeeding in *Acipenser naccarii* and *Oncorhynchus mykiss*. Thus, this present research revealed that starvation causes OS among juvenile largemouth bass, and refeeding results in regaining the initial values. Furthermore, to understand OS occurrence in the intestine of juvenile largemouth bass following starvation and refeeding, we investigated changes in the levels of Nrf2-Keap1 pathway components and the activities of endogenous antioxidant enzymes. Moreover, the Nrf2-Keap1 axis, as an important metabolic pathway, regulates both oxidative status and the immune response (Wang et al., 2024) [35]. Nrf2 modulates the transcription of antioxidant genes to deal with OS (Barcelos et al., 2016) [36]. Keap1 modulates the pathways by the ubiquitination and sequestration of most Nrf2 within the protoplasm. Consequently, only a tiny segment of Nrf2 is capable of entering the nucleus and interacting with antioxidant reaction elements among target genes, resulting in the stimulation of the expression of various antioxidative genes, such as *CAT*, *SOD*, *HO-1*, *GPx*, and *GCLC*. This eventually results in a decreased yield of reactive oxygen species (Feng et al., 2017; Du et al., 2020) [37,38]. Furthermore, the *Keap1* level was significantly downregulated, while the *Nrf2*, *HO-1*, *GPx*, and *GCLC* levels were significantly upregulated during starvation; these levels were restored after refeeding on day 7. These findings indicate that the cellular antioxidant defense system was activated by food restriction. This was experienced as OS at the cellular level. Thus, the current study shows that the Nrf2-Keap1 pathway was altered under starvation stress, indicating its function in stabilizing the impaired cell homeostasis, due to oxidative damage resulting from starvation.

Generally, OS activates the cellular protection mechanism and is tightly related to the inflammatory reaction, which is mediated by several factors such as IL-8, IL-10, IL-15, IL-1β, TNF-α, and TGF-β1 (Chen et al., 2015) [39]. IL-8, IL-15, IL-1β, and TNF-α are considered typical proinflammatory cytokines that induce host defense through an inflammatory reaction (Scapigliati et al., 2001) [40]. By comparison, IL-10 and TGF-β are vital anti-inflammatory cytokines restricting the inflammatory reaction via suppressing the release of proinflammatory cytokines, thereby restricting the overactivation of the immune response (Koj, 1998) [41]. Starvation can impair the immune status of fish and induce inflammation. For instance, starvation markedly raised the levels of *IL-1β*, *IL-6*, *NF-κB*, and *TNF-α* in *Megalobrama amblycephala* (Sun et al., 2020) [42]. Similarly, this research manifested that starvation increased the levels of *TNF-α*, *IL-1β*, and *IL-15* and reduced the levels of *IL-10* and *TGF-β*, thus suggesting that starvation adversely affects the immunologic function among largemouth bass guts, consistent with our previous findings in *Cyprinus carpio* L. (Zhao et al., 2022) [9].

As a physical barrier, the intestinal mucosa is extremely sensitive to food availability in fish species. During starvation, fish can change the intestinal mucosa via changing the surface region of brush borders and the dimensions of intestinal folds (German et al., 2010) [43], which directly disrupts the gut absorptive function and metabolic activity and adversely affects health and growth [44] (Ostaszewska et al., 2010). Specifically, starvation stress significantly decreased the mucosal fold height in the gut in *Salmo salar* L. (Baeverfjord and Krogdahl, 2010) [45] and reduced the surface area of *Pseudopleuronectes americanus* intestine (Mcleese and Moon, 2010) [46]. Our research uncovered that the microvillus height and mucosal folds of juvenile largemouth bass intestines were apparently decreased after starvation. The deterioration of microvilli in the intestine was directly related to reduced fold height and absorptive surface area, resulting in the destruction of intestinal physical barriers (Torrecillas et al., 2015) [47]. However, aquatic animals can adapt to poor food quality or deficiency by decreasing microvilli height and mucosal folds to sustain intestinal function (Park et al., 2008) [48]. Additionally, the impaired intestinal structure due to starvation stress resulted in a reduced absorption capacity of the intestine and decreased assimilation of nutrients in the gut, which might be associated with the induction of antioxidant defenses and inflammation, and a reduction in the activity of digestive enzymes. After refeeding, the dimensions of the mucosal folds and microvilli might be restored to their original levels as a partial compensatory effect. The number of GCs increased in the ST group, aligning with the discoveries for *Hippocampus erectus* (Su et al., 2022) [49]. This phenomenon is believed to be an adaptation to stress induced by starvation, as GCs are involved in the synthesis and secretion of mucin, which forms the protective barrier of epithelial cells (Elsabagh et al., 2018) [50].

The gut microbial communities are crucial for facilitating immunity and nutrient uptake within hosts (Xiong et al., 2015) [51]. Our research revealed that Acidobacteria, Actinobacteria, Bacteroidetes, Firmicutes, and Proteobacteria became the key phyla, and *Serratia and Lactobacillus* were the most dominant genera that markedly differed between the ST and RE groups, thus revealing that the microbiota population in juvenile largemouth bass intestines was impacted by food restriction. In addition, the abundance of diverse microorganism species markedly altered during starvation. Proteobacteria, which are commonly associated with energy regulation (Cuesta et al., 2022) [52], showed greater abundance in the RE group, thus suggesting that starvation caused energy deprivation in the microbiota, potentially leading to their death (Kohl et al., 2014) [53]. Bacteroidetes and Firmicutes are critical players in energy metabolism and the breakdown of undigested food through fermentation. Specifically, the F/B ratio is associated with constipation, as the members of these phyla process undigested food remnants and ferment polysaccharides (Birg et al., 2019) [54]. A marked relationship was observed between the rise in the F/B ratio and glucose metabolism, resulting in an elevation in energy absorption. This raises the potential for developing metabolic syndrome (Wang et al., 2019) [55]. Further, a more elevated F/B ratio was observed during the starvation period, which indicated that food restriction could induce an energy crisis and gut dysbiosis in microorganisms, subsequently leading to extensive microbial apoptosis (Kohl et al., 2014) [53]. Probiotic bacteria can improve the production of several antibacterial active peptides and metabolites to modulate the host gut health (González-Félix et al., 2018) [56]. Moreover, *Lactobacillus*, as a probiotic bacterial species, had a lower survival rate during starvation, in contrast to findings for *Oreochromis niloticus* (Sakyi et al., 2020) [57]. A possible difference between carnivorous largemouth bass and herbivorous *O. niloticus* is their respective feeding behaviors. Our findings demonstrate that starvation might effectively alter the probiotic bacterial population residing in the intestines of largemouth bass. In addition, the instability of intestinal microbial communities in juvenile largemouth bass could be linked to different pathways involving amino acid, energy, lipid, and carbohydrate metabolic functions that affect immunity. Thus, alterations in the gut microbiota composition can directly affect enzyme activities (Dai et al., 2018) [58], which subsequently affect metabolism and modulate the immune and growth characteristics of juvenile largemouth bass.

The intestinal microbiota generates various metabolites with critical effects upon the interplay between the intestine and the host. These metabolites can have positive and negative influences on the holistic health of the body (Nicolas et al., 2019) [59]. This study showed that analysis of the intestinal metabolite profile through metabonomics demonstrated that alterations in food restriction cause metabolic changes in largemouth bass. Mainly obtained from the degraded proteins, amino acids are pivotal players in numerous biological reactions and serve as materials to provide energy for several physiological processes (Wang et al., 2020) [60]. In the detection of differential metabolites, 17-octadecynoic acid, DG(20:3(5Z,8Z,11Z)/22:6(4Z,7Z,10Z,13Z,16Z,19Z)/0:0), 2′-deoxyguanosine-5′-monophosphate, (2R,3S)-piscidic acid, PE(16:1(9Z)/20:3(8Z,11Z,14Z)), and avocadyne 2-acetate were common differential metabolites among the three groups, thus indicating their participation in amino acid metabolism or their similarity to amino acid molecules. Consistent with this discovery, further elucidation by functional prediction analysis using 16S rRNA revealed a distinct correlation between amino acid metabolism and dietary restriction. This finding suggests that amino acid metabolism is disrupted during starvation in largemouth bass, while refeeding may ameliorate amino acid metabolism.

Moreover, our experimental results showed that guanosine, adenosine, and 2,6-dihydroxypurine, involved in purine metabolism, were enriched in the CON group compared to the ST group. Adenosine functions as a byproduct of purine metabolism and can enhance the resistance of isolated rat hearts to systemic ischemic arrest, which correlated with a reduction in the net degradation of ATP and an increase in ADP and AMP levels during the ischemic period. These findings indicate that purine metabolism plays a crucial role in maintaining ATP levels (Ely et al., 1985; Lasley, et al., 1988) [61,62]. ATP depletion leads to increased AMP, which is further transformed into deoxyinosine, inosine, guanosine, and xanthine and ultimately converted to uric acid, thereby increasing the levels of intermediates utilized to produce ATP (Sun et al., 2022) [63]. Significantly, our research indicates that starvation stress can hinder ATP production by reducing ADP levels and disrupting AMP in purine metabolism. This subsequently disrupts the energy metabolism of gut microorganisms in largemouth bass (Yao et al., 2024) [64]. In our view, this research is the first report under the current background.

## 4. Materials and Methods

### 4.1. Animals and Fish Welfare

Juvenile largemouth bass individuals were grown at the Huishui Commercial Farm Research Institute of Fisheries (Huishui, China) according to the following selection criteria: (1) high vigor and a capacity to feed; (2) common gray and black body coloration; (3) absence of symptoms such as rough fins, abdominal disruption, and hemorrhagic condition; (4) no obvious gut injury in histopathological evaluation. The fish in this study were managed in accordance with the reported recommendations (De Tolla et al., 1995) [65] and the established regulations by the Institutional Animal Ethics Committee.

### 4.2. Experimental Design and Management

Fish were reared in 10 tanks (200 L) with three times feeding daily on a commercially formulated diet (47.42% crude protein and 10.80% crude lipid on a dry-matter basis) to acclimate them to laboratory conditions. Subsequently, 270 fish (mean body weight [SEM]: 51.20 ± 0.18 g) were randomly allocated to three groups in triplicate; these individuals were randomly placed in 9 cylindrical tanks at the density of 30 fish per tank. The experiment lasted for 2 weeks; during this time 3 tanks of fish were fed three times daily (8:00, 12:00 and 17:00) with a commercial diet by using an automatic feeder (CON); while the fish in the 6 remaining tanks were starved (ST) during the first week. Immediately afterward, the previously fasted fish were refed for the remaining week (RE). The experimental setup was in accordance with Dar et al. (2019), who also starved the fingerlings of *L. rohita* up to 1 week and 2 weeks. A closed culture system was used. Purified subterranean water was used at a flow rate of 2 L/min. The quality of the water was monitored twice a week using a multi-parameter device (YSI, model 55-12FT, YSI Corporation, USA) to maintain an appropriate temperature (25.40 °C ± 1.00 °C), nitrogen level (0.03 ± 0.01 mg/L), dissolved oxygen level (7.75 ± 0.25 mg/L), and pH (7.00 ± 0.20).

### 4.3. Sample Collection

Three fish were sampled from each replicate at the completion of the experimental phases. After the induction of anesthesia with 150 mg/L of MS-222 (Sigma, St. Louis, MO, USA), a ventral cut was made in the fish while on ice. The gastrointestinal tract was dissected to obtain the proximal intestines, and the gut contents were placed immediately into 1.5 mL Eppendorf tubes and kept at −80 °C for in-depth analysis. For histological examination, the mid-intestine (2.0 cm) was fixed with 4% paraformaldehyde (PFA).

### 4.4. Digestive and Antioxidant Enzyme Activity Analysis

The intestinal tract was immediately extracted and pooled for the measurement of the digestive enzyme activities in fish from both the control and fasted-refed groups. Thus, each group included eight measurements for enzyme activities. Tissues were homogenized in four volumes (weight/volume) of ice-cold distilled water and centrifuged at 6000× *g* for 10 min at 4 °C. Then, the supernatant was harvested to measure the activities of digestive enzymes (amylase, trypsin, and lipase) using ELISA kits (Jiangsu Meimian Industrial Co., Ltd., Yancheng, China) following the manufacturer’s protocol. All enzymatic assays were performed within 12 h after the above treatment (Liu et al., 2020) [66].

Antioxidant enzyme activity was quantified using kits produced by Nanjing Jiancheng Bioengineering Institute (Nanjing, China) for malondialdehyde (MDA) content (Catalog No. A003-1-2), CAT (Catalog No. A007-1-1), and SOD (Catalog No. A001-3-2).

### 4.5. Histopathological Examination

Three intestinal samples from each group were treated with 10% formalin solution, and H&E staining was performed in accordance with Torrecillas et al. (2015). First, the tissue was rinsed with running water (10–20 min), and different concentrations of alcohol (from low to high) were used to displace water from the tissue. The sequence of treatments was as follows: 80% alcohol 1 times (once for 1–3 min), 95% alcohol (twice for 1–3 min), pure alcohol (twice for 5–10 min), xylene (twice). The tissues were then suspended in melted paraffin wax, embedded, and sliced into 3–5 μm sections. They were then strained with hematoxylin for 10 min, and after washing, differentiated with alcohol and hydrochloric acid. The tissues were then blueized with warm water, rinsed with running water for 5 min, and finally stained red with eosin for 20 s. The sections were made transparent with xylene and sealed with neutral gum. Observations were undertaken with a microscope (Nikon E100, Tokyo, Japan).

### 4.6. Quantitative Real-Time PCR Analysis

The total RNA was obtained via an RNAiso Plus kit (Takara Co., Ltd., Beijing, China); the concentration and quality of the extracted RNA were gauged via mass spectrophotometry and 1% agarose gel electrophoresis, separately. To be specific, RNA samples showing an A_260_/A_280_ proportion of 1.9–2.1 were selected. The RNA samples were reverse-transcribed to cDNA using the ExScript^TM^ RT-PCR kit (Takara Co., Ltd., Beijing, China) under the following conditions: 42 °C for 40 min, 90 °C for 2 min, and 4 °C for the remainder of the reaction. Table 3 shows the target primers used for the assays of *β-actin*, glutathione peroxidase (*GPx*), glutamate-cysteine ligase catalytic subunit (*GCLC*), heme oxygenase-1 (*HO-1*), interleukin-1β (*IL-1β*), *IL-8*, *IL-10*, *IL-15*, Kelch-like ECH-associated protein 1 (*Keap1*), nuclear factor erythroid 2-associated factor 2 (*Nrf2*), tumor necrosis factor (*TNF-α*), and transforming growth factor-β1 (*TGF-β1*).

Quantitative real-time PCR was performed through the ABI 7500 real-time PCR system integrated with the SYBR Premix Ex Taq^TM^ II (Tli RNaseH Plus) kit (Takara Co., Ltd.). The PCR reaction mixture contained 6 μL dH_2_O, 0.4 μL Rox Reference Dye, 10 μL SYBR Premix Ex Taq (2×), 2 μL RT reaction mix (cDNA solution), 0.8 μL PCR reverse primer (10 μM), and 0.8 μL PCR forward primer (10 μM). The PCR cycling conditions were listed below: an initial 10 s denaturing at 95 °C, followed by 45 cycles of 5 s denaturation at 95 °C, 15 s anneal at 62 °C, and 10 s extension at 72 °C, with careful plate reading at each step. A final 3-min extension was performed at 72 °C. β-Actin was selected as a reference for normalization. Gene expression was quantified using the 2^−ΔΔCT^ approach (Zheng et al., 2024) [67].

**Table 3:** Primers utilized for gene expression analysis by for qRT-PCR.

Genes	Primer Sequences F	Primer Sequences R	Size (bp)	Tm (°C)	Reference
*β-actin*	AAAGGGAAATCGTGCGTGAC	AAGGAAGGCTGGAAGAGGG	123	60	RNA-seq byXv et al. (2024) [68]
*IL-1β*	CAGCAGGCTCACAAAATAAACATCT	CGTGACTGACAGCAAAAAGAGG	234	60	RNA-seq by Lin et al. (2024) [69]
*IL-8*	CGTTGAACAGACTGGGAGAGATG	AGTGGGATGGCTTCATTATCTTGT	126	60	RNA-seq by Lin et al. (2024) [69]
*IL-10*	CGGCACAGAAATCCCAGAGC	CAGCAGGCTCACAAAATAAACATCT	122	59	RNA-seq by Lin et al. (2024) [69]
*IL-15*	GTATGCTGCTTCTGTGCCTGG	AGCGTCAGATTTCTCAATGGTGT	157	60	RNA-seq by Hu et al. (2023) [70]
*TNF-α*	CTTCGTCTACAGCCAGGCATCG	TTTGGCACACCGACCTCACC	104	60	RNA-seq by Hu et al. (2023) [70]
*TGF-β1*	GCTCAAAGAGAGCGAGGATG	TCCTCTACCATTCGCAATCC	186	59	RNA-seq by Hu et al. (2023) [70]
*Nrf2*	CAGACAGTTCCTTTGCAGGC	AGGGACAAAAGCTCCATCCA	150	60	RNA-seq by Hu et al. (2023) [70]
*Keap1*	CAGCTTACATGGCCGCATC	CTTCTCTGGGTCGTAAGACTCC	132	60	RNA-seq by Hu et al. (2023) [70]
*GPx*	CCCTGCAATCAGTTTGGACA	TTGGTTCAAAGCCATTCCCT	119	59	RNA-seq by Hu et al. (2023) [70]
*GCLC*	TACGGTGGCACGATGTCAGA	GGCAACCTAACCTTGGAAATG	194	60	RNA-seq by Zhao et al. (2022) [71]
*HO-1*	ATCGGAGCAGATTAAGGC	TTGTACTGTGGCAGGGTG	249	60	RNA-seq by Luo et al. (2023) [72]

Note: interleukin-1β *(IL-1β*), *IL-8*, *IL-10*, *IL-15*, tumor necrosis factor (*TNF-α*), transforming growth factor-β1 (*TGF-β1*), nuclear factor erythroid 2-related factor 2 (*Nrf2*), Kelch-like ECH-associated protein 1 (*Keap1*), glutathione peroxidase (*GPx*), glutamate-cysteine ligase catalytic subunit (*GCLC*), heme oxygenase-1 (*HO-1*).

### 4.7. 16S rRNA Gene Sequencing Analysis

The total DNA was obtained through the PowerSoil DNA Isolation kit (MO BIO Laboratories Inc., Carlsbad, CA, USA) as per the manufacturer’s specifications; and their quantity and quality were assessed at A_260_/A_230_ and A_260_/A_280_ ratios, respectively. The listed primers were applied t the PCR-based amplification of the V3 and V4 regions of the 16S rRNA gene: 338F: 5′-ACTCCTACGGGAGGCAGCA-3′ and 806R: 5′-ACTCCTACGGGAGGCAGCA-3′. The PCR products were decontaminated using the VAHTS^TM^ DNA Clean Beads (Vazyme Biotech Co., Ltd., Nanjing, China). Next, the products were quantified and standardized to produce a sequencing library. The library was precisely sequenced using the Illumina MiSeq platform (Illumina, San Diego, CA, USA) supplied by Biotree Biotechnology Co., Ltd. (Shanghai, China). The sequencing library was prepared using the TruSeq Nano DNA LT Library Prep kit (Illumina, San Diego, CA, USA) with utmost precision and care.

The FLASH and QIIME pipeline v1.8 were used to check the barcode sequences and data quality, and to delete the primer. Chimeric sequences were identified and removed using UCHIME. Operational taxonomic units (OTUs) were clustered using UPARSE v7.1 [version 7.1 http://drive5.com/uparse/ (accessed on 21 November 2023)] with a cutoff of 97% similarity. Next, the data were compared to the SILVA bacterial and archaeal databasev119 [version 119; http://www.arb-silva.de (accessed on 24 November 2023)] at the 70% confidence level, and the Ribosomal Database Project (RDP) classifier was used to assign the sequences to taxa. The community diversity [Shannon index: http://www.mothur.org/wiki/Shannon (accessed on 22 November 2023); Simpson index: http://www.mothur.org/wiki/Simpson (accessed on 20 November 2023)] and community richness [Chao 1 estimator: http://www.mothur.org/wiki/chao (accessed on 21 November 2023)]; ACE estimator: http://www.mothur.org/wiki/Ace (accessed on 25 November 2023) were analyzed using QIIME. Principal coordinate analysis (PCoA) was performed using the UniFrac online tool http://bmf.colorado.edu/unifrac (accessed on 25 November 2023). Functional characteristics of microbial communities were predicted using PICRUSt v1.0.0.

### 4.8. Metabolite Analysis

The gut contents of largemouth bass (50 mg) were subjected to careful extraction using 400 µL a methanol/water solution (4:1, *v*/*v*) to extract metabolites for the subsequent assay. The extracted metabolites were analyzed using an advanced Vanquish UHPLC system integrated with an Orbitrap Q Exactive^TM^ HF mass spectroscope (both from Thermo Fisher, Waltham, MA, USA), as described by Yu et al. (2022) [73]. Metabolite recognition was implemented using Progenesis QI data processing software 2.3, with original data construed through Progenesis QI version 2.3 (Waters Corporation, Shanghai, China). To perform comprehensive data analysis, R version 2.15.3 was utilized for tasks such as volcano plots, Venn diagrams, and principal component analysis (PCA); all these tasks were executed on the Majorbio Cloud Platform. Additionally, SIMCA 14.1 (Umetrics AB, Umea, Sweden) was used for further in-depth data analysis. Differential metabolites were precisely identified through a stringent selection process based on the following criteria: a fold-change > 1.0 or <1.0, a VIP score > 1.0, and a *p* threshold of <0.05, indicating statistical significance. This rigorous analysis was conducted using resources such as LIPIDMAPs and involved thorough exploration of the KEGG and HMDB databases for comprehensive metabolite characterization.

### 4.9. Statistical Analysis

The data were denoted as the mean ± standard error of the mean (SEM). The data were statistically analyzed using Duncan’s multiple range test with SPSS 24.0 (IBM Corp, Armonk, NY, USA). A one-way analysis of variance was conducted on the entire dataset. Before analysis, the normal distribution was assessed using the Shapiro–Wilk test. Further, the variance homogeneity was assessed through Levene’s test. A *p*-value of <0.05 was considered statistically significance.

Moreover, Spearman rank correlation analysis was performed on the differences in species abundance among samples in order to identify data with a correlation coefficient greater than 0.1 and a *p*-value smaller than 0.05, which were used to construct the network. This network analysis is designed to obtain coexistance relationships among species in environmental samples, interactions among species within the same environmental condition, which further helps reveal the mechanisms of differential phenotypes between samples.

## 5. Conclusions

**Figure 10:**
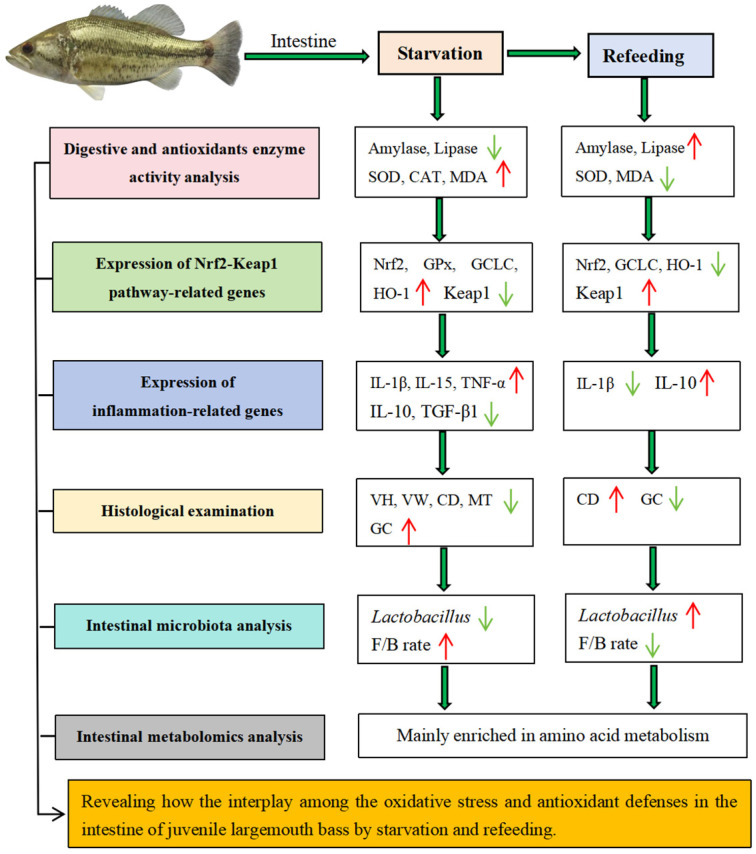
Intestinal microbial and immune responses of juvenile largemouth bass after the onset of starvation and refeeding. Green and red arrows indicate decreased and increased responses, respectively. (For the interpretation of the references to color in this figure legend, the reader is referred to the web version of this article).

The present study aimed to describe the interplay between antioxidant defenses and OS in the intestine of juvenile largemouth bass following starvation and refeeding, encompassing factors in intestinal digestion, intestinal structure, the antioxidant system, inflammation, and the function of the microbiota (Figure 10). Starvation stress might induce greater susceptibility to pathogen infection by lowering the survival rate of potential probiotics, destroying the intestinal structure, increasing inflammatory responses, and causing energy metabolism disorders in the gut microbial population. Refeeding enhances the microbial community structure of fish intestines by increasing the ratio of beneficial bacteria and reducing OS-induced damage, thereby enabling the achievement of the initial values compared to those of the control. These findings deepen our comprehension of the intestinal health of juvenile largemouth bass in coping with starvation and refeeding, thus contributing to enhancing the feeding strategies of fish.

## Figures and Tables

**Table 1:** Information on 16S rRNA sequencing of different gut flora groups in juvenile largemouth bass.

Sample	Raw Reads	Clean Reads	Effective Reads	Average Length/bp	Effective Rates/%	Q30 %
CON	79,959	79,632	75,819	419	92.80	93.61
ST	79,813	79,497	74,684	419	93.58	93.58
RE	79,968	79,648	75,523	420	94.44	94.44
Total	239,740	238,777	226,026	—	—	—
Average	—	—	—	419	93.61	93.88

**Table 2:** Number of sequences analyzed, estimated community richness index, and community diversity index for 16S RNA of gut microbiota in juvenile largemouth bass.

Item	ACE	Chao1	Simpson	Shannon
CON	1881.61 ± 230.34	1538.03 ± 62.23 ^a^	0.98 ± 0.00	8.13 ± 0.18 ^b^
ST	1904.12 ± 342.81	1432.48 ± 150.30 ^ab^	0.98 ± 0.00	8.91 ± 0.13 ^a^
RE	1941.44 ± 396.26	1172.01 ± 48.87 ^b^	0.99 ± 0.00	8.15 ± 0.07 ^b^

Significant differences between the different conditions are identified with different marked letters (*p* < 0.05).

## Data Availability

Data is contained within the article.
